# Duration of bed occupancy as calculated at a random chosen day in an acute care ward. Implications for the use of scarce resources in psychiatric care

**DOI:** 10.1186/1744-859X-4-11

**Published:** 2005-05-27

**Authors:** John E Berg, Asbjørn Restan

**Affiliations:** 1 Lovisenberg Diaconal Hospital 0440 Oslo, Norway; 2 Akershus University Hospital Clinic of Psychiatry 1484 Lørenskog, Norway

**Keywords:** Psychiatry, resident treatment, cost-effectiveness, treatment logistics

## Abstract

**Background:**

Psychiatric acute wards are obliged to admit patients without delay according to the Act on Compulsive Psychiatric Care. Residential long term treatment facilities and rehabilitation facilities may use a waiting list. Patients, who may not be discharged from the acute ward or should not wait there, then occupy acute ward beds.

**Materials and methods:**

Bed occupancy in one acute ward at a random day in 2002 was registered (n = 23). Successively, the length of stay of all patients was registered, together with information on waiting time after a decision was made on further treatment needs. Eleven patients waited for further resident treatment. The running cost of stay was calculated for the acute ward and in the different resident follow-up facilities. Twenty-three patients consumed a total of 776 resident days. 425 (54.8%) of these were waiting days. Patients waited up to 86 days.

**Results:**

Total cost of treatment was 0.69 million Euro (0.90 mill. $), waiting costs were 54.8% of this, 0.38 million Euro (0.50 million $). The difference between acute care costs and the costs in the relevant secondary resident facility was defined as the imputed loss. Net loss by waiting was 0.20 million Euro (0.26 million $) or 28.8% of total cost.

**Discussion:**

This point estimate study indicates that treating patients too sick to be released to anything less than some other intramural facility locks a sizable amount of the resources of a psychiatric acute ward. The method used minimized the chance of financially biased treatment decisions. Costs of frustration to staff and family members, and delayed effect of treatment was set to zero. Direct extrapolation to costs per year is not warranted, but it is suggested that our findings would be comparable to other acute wards as well. The study shows how participant observation and cost effectiveness analysis may be combined.

## Background

Treatment of acute psychiatric illness in resident settings is expensive, albeit less so than in intensive medical care units [1-3]. The costs are to a great extent indispensable, but not of the same magnitude in all elements of the treatment chain. Allocation of patients in a treatment and cost-efficient way along this chain is a logistic challenge. Regression-based cost functions have been used to illustrate patient and system related cost elements[4]. The possibility of violence in acute psychosis is an important contributor to the costs[5]. Psychiatric acute wards in Norway are obliged by law, the Norwegian Act on Compulsive Psychiatric Care (ACPC), to accept persons who, after an examination by an external doctor are found to be in danger of severely damaging own life or other people's lives. Reasons for referrals are acute psychosis, mania, or severe suicidal conditions. A person may be referred voluntarily to a mental hospital, or for compulsory observation of up to 10 days or for compulsory treatment for a prolonged period of time. Not later than 24 hours after admission the consultant psychiatrist on duty has to make a legally binding decision on admission status. The patient or his relatives may appeal this decision to a legal body outside the hospital.

The acute wards are often the first step in a chain of facilities that the patient may need in order to regain his functional ability. Such secondary facilities, sub-acute/intermediate wards, long term treatment, half-way houses or nursing care homes are, however, not obliged to accept patients at demand, but rather as empty places/beds become available. The result may be crowded acute wards. Either because too many patients are referred to the wards per time unit or because patients do not get another place to stay, if they are too sick to be transferred to community services of ambulatory type.

Shortened duration of resident stays might be cost-effective treatment, although the clinical outcome may be variable. High rates of relapse may be counterproductive. In a Norwegian study relapses were shown not to be an indicator of efficiency, but rather of logistic planning of mental health services[6].

Waiting time whilst in resident acute care has not, to our knowledge, been studied from a clinical and economic standpoint using cross-sectional data.

The decision on length of stay was taken on daily meetings of the treatment staff, where all the doctors, psychologist and social workers were present. The group of senior psychiatrists decided where the patient should be referred after acute care treatment. If no resident place was available at the desired time, the patient had to wait in the acute ward for such a slot.

The aim of the study was thus to present a novel way of calculating the partial cost efficiency of waiting time in one of four acute wards in the city of Oslo (ca 500.000 inhabitants).

## Methods

**Figure 1:**
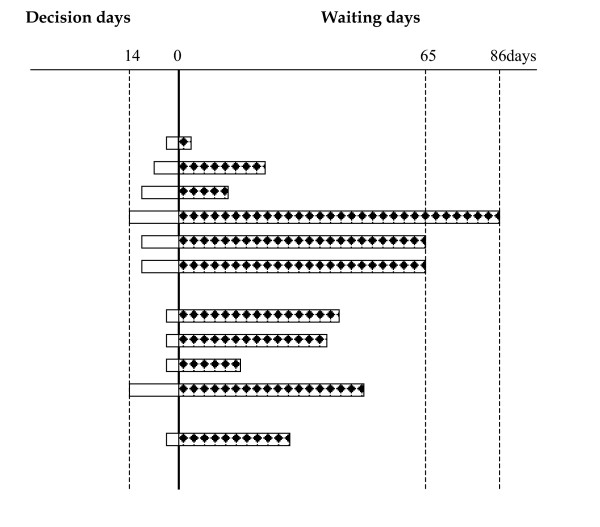
Number of resident days for 11 out of 23 patients in an acute ward before a decision was made for further intramural treatment in a less costly facility, and number of waiting days after the decision day. Twelve patients did not wait for other intramural treatment, and did not accumulate waiting days.

All resident patients (N = 23) on a randomly chosen day in March 2002 were included. Day of entry, which was different from the chosen day, legal admission status, sex, age and number of previous resident stays were noted, see table 1 and figure 1.

**Table 1:** Socioeconomic data and level of compulsion according to ACPC* by entry for 23 patients who all were inpatients on a random chosen day in 2003 in the acute wards of a psychiatric hospital, (Standard deviation).

	*Waiting for further treatment*	*Not waiting*
	
	*(N = 11)*	*(N = 12)*
Men	7	5
Women	4	7
		
Mean number of earlier referrals	3.6 (3.2)	5.7 (6.2)
		
Mean age	34.4 (10.4)	36.8 (8.6)
		
Psychotic illness	11 (47.8%)	8 (34.8%)
		
§according to Law:		
§2-1 (voluntarily)	1	3
§3-6 (compulsory observation)	5	3
§3-3 (compulsion)	5	6
		
§as decided by senior psychiatrist within 24 hours after admission:		
§2-1	1	3
§3-6	3	3
§3-3	7	6

*) ACPC = The Norwegian Act on Compulsory Psychiatric Care of 1999.

Only one of the authors, JEB, a psychiatrist in training at the time of the study, was aware of the registration of time from the senior psychiatrists decision to the actual day of referral to the next step in the treatment chain, called decision days in figure 1. Waiting days were defined as the number of days from this decision date to the factual transfer. Patients were initially evaluated for some days before such a decision would be made. One patient was still waiting at study end. Waiting time for this patient was truncated at the end of the study period.

### Cost estimation

Direct costs of treatment were calculated as the daily inpatient expenditures multiplied by number of days. The same costing procedure was used for cost of stay per day in the different secondary facilities. Cost of waiting in the acute ward was then calculated as the difference between cost of residency in the acute ward and the chosen secondary facility. This cost was withdrawn from the cost of the acute ward stay for each patient. The costs used in the calculations were taken from the hospital and secondary facility balances of 2001.

## Results

Twenty-three patients were in the acute wards at the chosen day. Twelve were men (52.2%) and 11 (47.8%) women. Mean age was 35.7 (SD = 9.3) with a range from 22 to 56, table 1. Four patients were referred voluntarily to the acute ward, 8 were under compulsory observation, and 11 under compulsion for a prolonged period.

Eleven patients (47.8%) waited for secondary resident treatment. All patients waiting for further intramural treatment suffered from a psychotic illness, whereas 8 of 12 not waiting had a psychotic illness.

Duration of stay was composed of the days preceding the chosen day and the number of days of further treatment in the acute ward. Twenty-three patients had a total of 776 days, of which 425 (54.8 %) were waiting days as defined above. Waiting time for single patients varied from one day to 86 days. There were altogether 7925 resident days in the wards during the year 2001.

Net running costs for the acute wards that year were 7,001,960 Euro (9,156,410 $). Spurious incomes of 394,853 Euro (516,346 $) and the acute ward's share of the food, cleaning, and computer services costs of the whole general hospital were not included.

Net running costs of the largest secondary facility were

8,095,343 Euro (10,586,217 $). The number of resident days were, respectively, 7919 and 14187 during 2001. Mean cost per day of treatment was 884 Euro (1156 $) and 570 Euro (746 $), respectively for the acute wards and the largest secondary facility. The cheapest secondary facility had a cost per patient day of 168 Euro (219 $).

Table 2 gives the percentage of patients waiting at randomly chosen days from each of ten other months of 2002. From 9.5% to 39.1% waited on the chosen days.

**Table 2:** Patients referred to the acute wards during the first ten months of 2002 according to waiting status (%) and level of compulsion according to the ACPC.

Month	Waiting (%)			Level of compulsion (%)		
	*Yes*	*No*		*§2-1 Voluntary treatment*	*§3-6 Compulsory observation*	*§3-3 Compulsory treatment*
1	31.0	69.0		44.8	6.9	48.3
2	39.1	60.9		39.1	13.0	47.8
3	33.3	66.7		25.0	16.7	58,3
4	16.7	83.3		50.0	12.5	37.5
5	25.0	75.0		53.1	21.9	25.0
6	29.2	70.8		37.5	12.5	50.0
7	28.6	71.4		46.4	10.7	42.9
8	9.5	90.5		52.4	19.0	286
9	34.8	65.2		52.2	8.7	39.1
10	27.6	72.4		31.0	10.3	58.6

Mean age varied from 34.9% (SD = 8,0) to 39.4% (SD = 10,1) on the chosen days. For those waiting mean age was 34.0 (SD = 9.0) and for those not waiting for further intramural treatment 38.0 (SD = 11.2).

Net running cost was 686,132 Euro (897,250 $) and the amount allocated to waiting 375,781 Euro (491,406 $), i.e. 54.8 %. The net loss accrued in the acute wards was 197,693 Euro (258,522 $), and constitutes 28.8 % of net running cost, i.e. the difference between waiting cost at the acute wards because of delayed further referrals and the net running cost at the secondary facilities.

## Discussion

The impression of many clinicians that patients are waiting unnecessarily in the acute wards is confirmed by the present study. The net loss to the chain of treatment facilities, regardless of where the loss is incurred, was 28.8% of total net running costs, as calculated for the 425 waiting days in resident treatment. A financial system exists that does not contribute to make these costs explicit. Neither the acute wards nor the secondary resident facilities were made economically responsible for the imputed loss. Cost containment would be attained more easily if the economic responsibility covered the complete chain of facilities. That would also give more efficient logistics of patients through the treatment chain.

Patients had to wait in the acute ward because referral to ambulatory treatment or treatment at home was deemed clinically irresponsible by the senior psychiatrists.

As shown in table 2, the choice of one day in March represented a higher percentage of waiting patients than post hoc observed for the rest of the year. Length of waiting is, however, not causally related to number of waiting patients, but to factors inherent in the logistics of the psychiatric treatment sector.

We have not tried to aggregate our results to an effect for a longer period of time, as this would demand more data on both costs and incomes to the whole treatment chain [7,8]. The present study was not set up to comply with such a comprehensive cost utility or cost effectiveness analysis.

Treatment planning for schizophrenia patients after hospitalisation and into more permanent care outside the hospital has been difficult to standardise, but studies show that this would be of importance [9,10]. Early intervention in recently diagnosed schizophrenia has been advocated as a method to reduce future loss of functional abilities[11]. Early intervention is aimed at reducing duration of untreated psychosis, DUP time. Successful reduction of DUP time is also suggested to reduce costs of long-term treatment. Patients with substantial psychosocial problems are also frequent users of resident treatment[12]. The difference between ambulatory and resident psychiatric care was studied by Creed etal in a randomised controlled trial of 179 patients deemed to profit from either[1]. The authors found, as expected, that day treatment was cheaper than inpatient treatment for those patients who could be in day treatment. Inpatients improved significantly faster, but at 12 months the burden on families, also the economic burden, was equal in the two groups. Deinstitutionalisation has been studied in a cost effectiveness perspective[13]. Cost of treatment was lower in long-term patients discharged from hospital compared to those staying. Released patients turned out to be healthier along several dimensions of positive health.

Patients in need of continued care are often best helped by treatment and rehabilitation efforts close to where they live. Such care can be sufficient and appropriate after acute care, but a priori it is not necessarily cost effective. If this really is the case, it would be even more important to use acute care resources in an efficient way, i.e. delivering the services needed at the right time in the right facility.

Waiting time is not per se a waist of money [14]. Zero waiting time requires excess capacity, probably higher than necessary from a public point of view. The American health financial system is organised with a tilt towards shorter waiting times, and thus towards higher expenditure per capita. Waiting time should be the result of clinical judgments, not bad logistics.

Direct costs, as daily inpatient expenditures multiplied by number of days, were used in a study of waiting time before relapse in schizophrenia in USA. Indirect costs of treatment were not estimated[15]. A similar approach, disregarding other ancillary costs, was used in the present study. Cost of illness analysis estimates running and maintenance costs as direct costs, and cost of mortality and morbidity as indirect costs[2,16]. Dorothy Rice found that the direct costs of schizophrenia and affective disorders were twice the indirect costs. The share of the costs of these two illnesses was higher than the prevalence would suggest. However, cost of illness analysis tends to give huge cost estimates, disregarding any imputed gain for others.

One group of patients was not considered as waiting in the present study. Several of our patients are referred to the acute ward partly because they also are homeless, and they often get their stay prolonged for social reasons. Homelessness is partly a social welfare problem, but might also be a corollary to the incumbent loosing his home because of psychotic acts (fires, non-payment of rent, destructive and disturbing behaviour).

The patients waiting in the acute wards used the services of the department to the same extent as the other patients, they were not "idly waiting". Some established a close therapeutic relationship to therapists in the acute ward, who later could not follow up the patient due to other tasks. This may be detrimental to some patients, and could have been avoided if referrals to secondary institutions were smoother. The burden on the families would also be increased by the uncertainty[17]. These factors are not part of the calculations of the study, but would if entered have increased the imputed loss. In a study of re-entries, half the patients had previously been resident patients[6]. Fewer re-entries were observed in patients with long and planned stays, sufficiently organised end of resident stay and follow-up visits. The amount of follow-up by community centres did not improve outcome. Lack of beds in the acute wards due to waiting also affects the health of those seeking treatment, as they would have to wait longer and get their mental status deteriorated.

The impact of waiting on staff performance and therapeutic efforts has not been studied here, but would perhaps also be of importance for sick leave and burnout.

It is probably a questionable option to shorten resident stays in the acute wards without augmenting the quantity and/or quality of services outside the ward, but it would partly solve the lack of beds, before re-entries increases.

Cost of new antipsychotic medication has been used as an argument for cost containment. But at Norwegian prices for medication, even the most expensive atypical antipsychotics in relevant doses for a patient year would not cost more than 7–8 days of resident treatment [18,19].

Waiting for treatment may be rational. Absolutely no waiting for entry to a facility in the treatment chain would demand a capacity which would not be used cost efficiently, the case observed for instance in the airline business. If waiting is necessary, we should avoid waiting at the most expensive slots in the chain, i.e. in acute wards for psychiatric treatment[14]. The financial system of public health care treatment chains makes it difficult to discover where the financial loss accrues, as the acute wards in this case do not take regress on the extra expenses at the following secondary facility not offering a treatment option at the desired time point.

The referring doctors outside the acute ward find it increasingly difficult to send patients to acute care, and the number of compulsory referrals is high, in this study 19 out of 23 patients. This would probably represent an economic burden on the families of severely ill psychiatric patients in the case of unduly delayed referrals[17]. The costs demonstrated in the present study should therefore be viewed as a minimum estimate. Modern psychiatric treatment should thus be given the possibility to use the given economic and clinical resources in a cost effective way. This would also include care given by municipalities and private contributors.

## Conclusion

A substantial part of the costs of running an acute psychiatric ward, 29% of running costs in this study, were allocated to waiting. Better logistics in the treatment chain could change this, and several economic incentives along the chain could be used. A treatment chain were only one link is obliged to accept patients without delay, would probably not be the ideal solution. This study indicates that participant observation and cost effectiveness analysis may be combined.

## Conflict of interest

Both authors were salaried workers in the facility at the time of the study. No financial or other conflicts of interest were present.
